# Serial CSF CA19–9 monitoring and CSF genomic profiling in ERBB2-mutant lung adenocarcinoma with leptomeningeal metastasis: a case report

**DOI:** 10.3389/fonc.2026.1925627

**Published:** 2026-08-18

**Authors:** Qilin Wang, Jing Wen, Beilei Zeng, Chuanyu You, Xinyu Chen, Juncai Ye, Tian Xu, Xueping Yang, Jiahao Chen, Guangzeng Zhou, Dongsheng Yuan, Yan Gui

**Affiliations:** 1 Department of Oncology, Affiliated Hospital of North Sichuan Medical College, Nanchong, China; 2 North Sichuan Medical College, Nanchong, China; 3 Key Laboratory of Integrated Precision Diagnosis and Treatment for Solid Tumors, Sichuan Provincial Health Commission, Nanchong, China

**Keywords:** CA19-9, ERBB2/HER2, intrathecal chemotherapy, leptomeningeal metastasis (LM), lung adenocarcinoma

## Abstract

Leptomeningeal metastasis (LM) from ERBB2-mutant lung adenocarcinoma is difficult to treat and monitor because systemic disease and leptomeningeal disease may evolve discordantly. Evidence regarding cerebrospinal fluid (CSF) genomic profiling and serial CSF tumor marker monitoring in ERBB2-mutant non-small cell lung cancer with LM remains limited. We report a 48-year-old woman initially diagnosed with stage IB mucinous lung adenocarcinoma harboring an ERBB2 exon 20 p.G776delinsVC mutation. After surgery and adjuvant chemotherapy, she developed nodal recurrence and later presented with lower back pain and lower-limb numbness. LM was clinically diagnosed based on neurological symptoms, magnetic resonance imaging and CSF cytopathology. She received craniospinal irradiation, systemic therapy and subsequent intrathecal treatment. At month 33, CSF CA19–9 was markedly elevated despite no clear radiographic systemic progression. CSF next-generation sequencing(NGS) detected the same ERBB2 exon 20 p.G776delinsVC mutation as the primary lung tumor, with a higher variant allele fraction in CSF than in lung tissue.The patient subsequently received trastuzumab deruxtecan and sequential intrathecal therapy with pemetrexed, etoposide, cytarabine and pemetrexed rechallenge. Intrathecal treatment was adjusted according to serial CSF CA19–9 levels, neurological status, imaging findings, systemic disease activity and treatment-related toxicities. CSF CA19–9 was not used as a stand-alone criterion for progression or treatment modification.At the latest follow-up, she remained alive more than 36 months after the clinical diagnosis of LM. This case suggests that CSF NGS and serial CSF CA19–9 may provide further insights into disease activity within the integrated assessment of systemic and leptomeningeal disease. Their clinical applicability is still under investigation and necessitates future validation.

## 1 Introduction

Lung cancer is one of the most often diagnosed cancers globally and is a primary cause of cancer-related deaths, with non-small cell lung cancer (NSCLC) representing around 85% of all lung cancer cases (1).Human Epidermal Growth Factor Receptor 2(HER2) mutations are infrequent in lung adenocarcinoma, present in roughly 2–4% of cases, and are more commonly found in women and never-smokers (2). Recent molecular studies have further highlighted the biological heterogeneity of lung adenocarcinoma, including transcriptional programs such as PHLDA2/ELK1 signaling that may promote malignant progression (3).

The treatment landscape for ERBB2-mutant NSCLC has progressed swiftly. Trastuzumab deruxtecan, an antibody-drug combination(ADC) targeting HER2, has shown significant systemic efficacy in ERBB2-mutant NSCLC patients who have undergone prior treatment, along with indications of intracranial effectiveness in certain individuals with brain metastases. Selective HER2 tyrosine kinase inhibitors (TKIs), such as zongertinib and sevabertinib, have exhibited encouraging efficacy, whereas previous medicines like poziotinib and pyrotinib displayed anticancer effects but were constrained by toxicity and a lack of substantial CNS-specific evidence (4–8). However, data concerning the effectiveness of HER2-targeted treatments in active LM is limited. Interventions targeting parenchymal brain metastases should not be directly applied to LM due to variations in anatomical distribution, tumor biology, and pharmacokinetics within the leptomeningeal and cerebrospinal fluid(CSF) environments. Consequently, the ideal systemic and intrathecal therapeutic approaches for ERBB2-mutant NSCLC with LM are yet to be established.

LM is a significant late-stage consequence of lung cancer, with prior studies indicating a median overall survival of roughly 10.9 months following the onset of LM (9). LM encompasses the dissemination of tumors throughout the leptomeninges and the subarachnoid area filled with CSF. Systemic treatments need to penetrate the blood-brain or blood-CSF barriers, while intrathecal administration introduces medications directly into the CSF. However, effectiveness can still be constrained by compromised CSF circulation and inadequate infiltration into substantial or nodular masses (10).Intrathecal chemotherapy(ITC) can enhance drug concentration in the CSF, and intrathecal pemetrexed has demonstrated promising efficacy in LM from lung cancer; however, evidence for non-standard intrathecal drugs, such as etoposide, is limited (11, 12).

Here, we report a patient with ERBB2-mutant mucinous lung adenocarcinoma who developed LM and received trastuzumab deruxtecan and sequential ITC as part of the integrated management of systemic and leptomeningeal disease.The intrathecal protocol was systematically modified based on sequential CSF CA19–9 concentrations and treatment-associated toxicities. The patient has survived for over 36 months following the clinical diagnosis of LM and the commencement of treatment, and continues to be monitored. This report is intended to describe an exploratory approach to longitudinal CSF monitoring rather than to establish CSF CA19–9 as a validated biomarker.

## 2 Case presentation

### 2.1 Patient history and examinations

**Figure 1:**
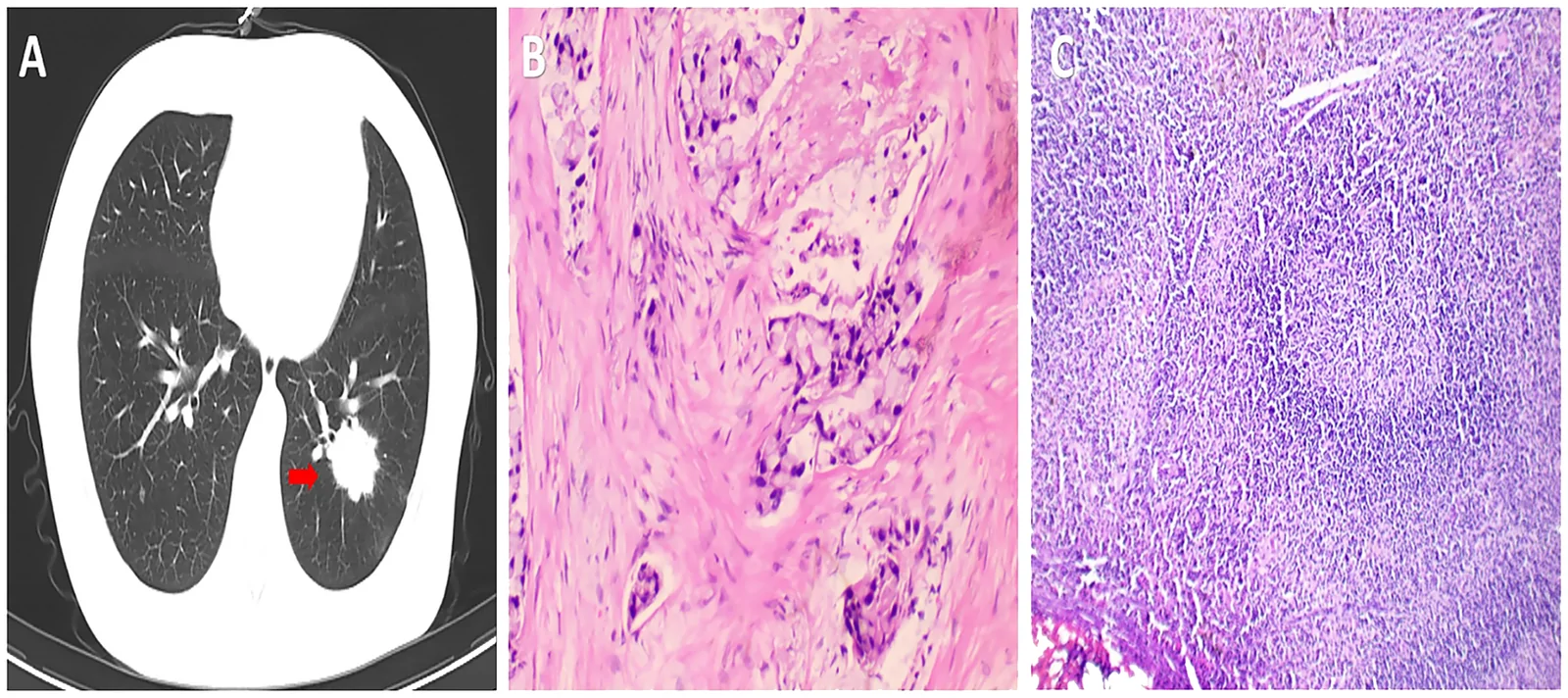
Representative baseline images of the patient. **(A)** Chest CT showed a 3.2 cm × 2.6 cm mass in the lateral basal segment of the left lower lobe. **(B)** Representative histopathological image of the primary tumor. **(C)** Histopathological examination of the lymph nodes showed no evidence of metastatic carcinoma.

A 48-year-old woman arrived at our hospital on November 2021(Month1), with a two-week history of a left lung mass identified during a regular physical examination. She had no prior history of hypertension, diabetes mellitus, tobacco use, or alcohol intake, and her Eastern Cooperative Oncology Group(ECOG) performance status was 1. Chest CT revealed a 3.2 cm × 2.6 cm lesion in the lateral basal segment of the left lower lobe (**Figure 1A**). Ultrasonography of cervical lymph nodes, whole-body bone scintigraphy, and brain magnetic resonance imaging(MRI) revealed no abnormalities. In the 1st month, the patient underwent video-assisted thoracoscopic left lower lobectomy accompanied by intrathoracic lymph node dissection. The postoperative pathological assessment verified mucinous adenocarcinoma in the left lower lobe, with no metastatic carcinoma detected in the analyzed lymph nodes (**Figures 1B, C**). Next-generation sequencing(NGS) detected an ERBB2 exon 20 p.G776delinsVC mutation, with a variant allele fraction of 19.42%. Programmed death-ligand 1(PD-L1) immunohistochemistry was performed using the 22C3 assay. PD-L1 expression showed a tumor proportion score(TPS) of <1% and a combined positive score(CPS) of 5. The patient was diagnosed with stage IB lung adenocarcinoma (pT2aN0M0) according the eighth edition of the American Joint Committee on Cancer(AJCC) staging system. She subsequently received four cycles of adjuvant chemotherapy with nedaplatin and pemetrexed.

**Figure 2:**
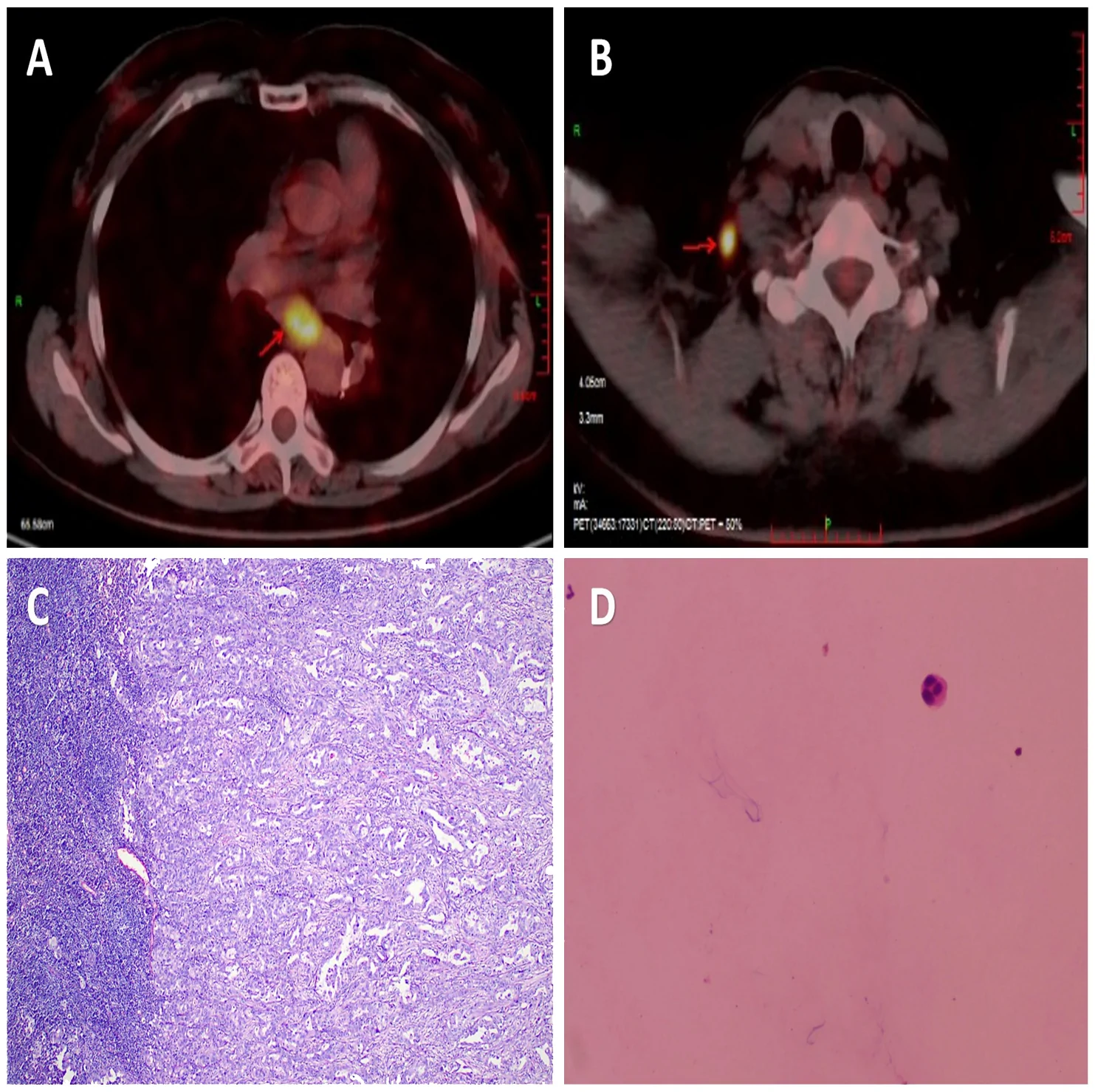
Representative images during treatment. **(A)** PET-CT image showing increased fluorodeoxyglucose uptake in the subcarinal lymph node. **(B)** PET-CT image showing increased fluorodeoxyglucose uptake in the right supraclavicular lymph node. **(C)** Histopathological image of metastatic adenocarcinoma in a lymph node.**(D)** CSF cytology showing atypical cells. Lesions are indicated by red arrows. PET-CT=Positron emission tomography/computed tomography. CSF, cerebrospinal fluid.

In the 9th month, subsequent testing indicated an increased serum carcinoembryonic antigen(CEA) level. Positron emission tomography/computed tomography(PET-CT) revealed enlargement of the right supraclavicular and subcarinal lymph nodes with elevated fluorodeoxyglucose uptake (**Figures 2A, B**). A subsequent needle biopsy of a cervical lymph node confirmed the presence of metastatic cancer (**Figure 2C**). The metastatic lesions were addressed with concurrent chemoradiotherapy, delivering 66 Gy in 33 fractions to the planned gross tumor volume, with concurrent chemotherapy utilizing an EP (etoposide and cisplatin) regimen. She then received maintenance durvalumab, achieving a partial response (PR) as evaluated by the Response Evaluation Criteria in Solid Tumors version 1.1.(RECIST1.1)

### 2.2 Therapeutic intervention

At month 19, the patient developed lower back pain and lower-limb numbness. Brain MRI revealed suspicious punctate leptomeningeal enhancement, and CSF cytology obtained via lumbar puncture showed atypical cells (**Figure 2D**). MRI of the spine demonstrated multiple enhancing nodules along the cervical and lumbar spine and within the spinal leptomeningeal space. Based on these findings, the patient was clinically diagnosed with LM. After multidisciplinary discussion, the patient received craniospinal irradiation, initially planned as 36 Gy in 20 fractions but reduced to 30.6 Gy in 17 fractions because of grade 3 myelosuppression.

During subsequent treatment, the financial burden associated with continued durvalumab therapy became a major concern for the patient. After discussion of treatment affordability, accessibility, potential benefits, the systemic therapy between the month 21 to the month 32 was altered to bevacizumab, pemetrexed, and cisplatin, resulting in a PR. In the 33rd month, radiographic evaluation indicated no signs of disease development; nevertheless, CSF CA19–9 levels were significantly higher at 34,628.4 U/mL, while serum CA19–9 levels were 2,949.5 U/mL, indicating possible heightened activity of the leptomeningeal disease. Concurrent CSF NGS identified the ERBB2 exon 20 p.G776delinsVC mutation, with a variant allele fraction of 54.26%. The patient then underwent ITC of pemetrexed at a dosage of 30 mg weekly via lumbar puncture, and systemic therapy was modified to include bevacizumab, sintilimab, and pemetrexed.In the 35th month, according the Common Terminology Criteria for Adverse Events version(CTCAE) 5.0, the patient had treatment-related hematologic and hepatic toxicities, comprising grade 3 leukopenia, grade 3 thrombocytopenia, and grade 2 hepatic dysfunction. The intrathecal pemetrexed dosage was consequently reduced to 20 mg weekly, followed by a decrease in CSF CA19–9 levels.

**Figure 3:**
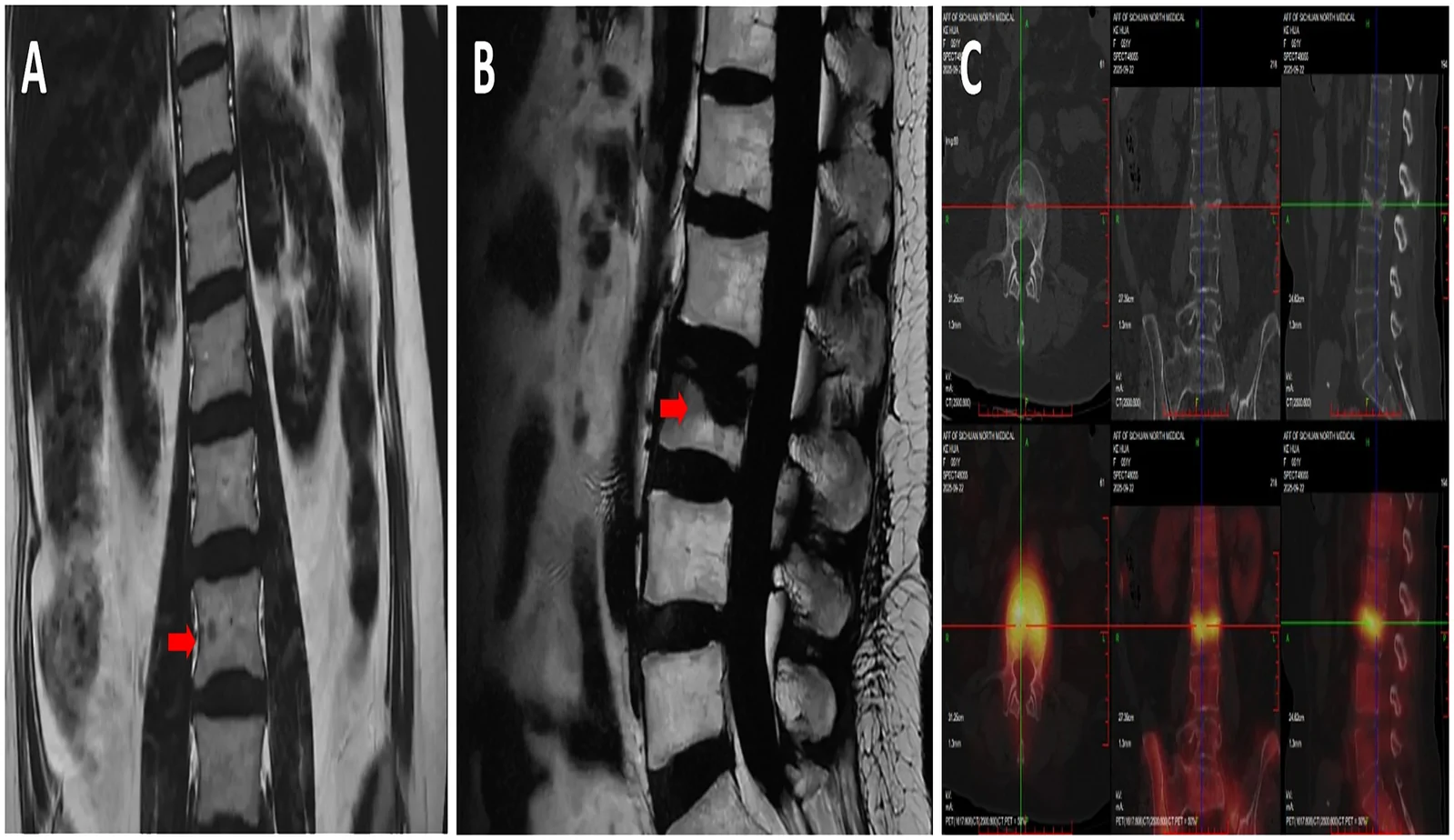
Representative imaging findings during treatment. **(A)** Lumbar spine MRI showed multiple enhancing nodules along the lumbar spine and in the submeningeal space. **(B)** Lumbar spine MRI showed a focal depression at the superior endplate of the L3 vertebral body. **(C)** SPECT-CT Image.SPECT-CT showed abnormal radiotracer uptake in the L3 vertebral body. Lesions are indicated by red arrows. MRI=magnetic resonance imaging. SPECT-CT, single photon emission computed tomography.

Due to inadequate tolerance to many lumbar punctures, the patient received an Ommaya reservoir implantation for intraventricular medication delivery in the 39th month. The systemic therapy was simultaneously transitioned to trastuzumab deruxtecan at a dosage of 5.4 mg/kg every three weeks. Due to myelosuppression, the ITC protocol was modified to etoposide, with an induction schedule of 0.5 mg administered on days 1–5 weekly for 8 weeks, followed by maintenance therapy at 0.5 mg on days 1–5 every 4 weeks. Throughout the treatment, CSF CA19–9 concentrations persisted in declining. At Month 47, a new L3 vertebral metastasis was identified, and the overall disease status was classified as progressive disease(PD) due to this new extraleptomeningeal lesion.Serum and CSF CA19–9 levels also increased; the biomarker increases raised concern for possible leptomeningeal disease activity but were not considered sufficient to establish LM progression. (**Figures 3A–C**). The ITC regimen was modified to cytarabine at a dosage of 30 mg biweekly, and the L3 spinal lesion was addressed with palliative irradiation at 24 Gy delivered in 6 fractions. In January 2026, a follow-up evaluation indicated stable illness. At month 51, CSF CA19–9 levels significantly elevated without radiographic development, leading to an intrathecal pemetrexed rechallenge utilizing an induction regimen of 20 mg on days 1 and 4 weekly for 3 weeks, succeeded by maintenance therapy at 20 mg on day 1 every 3 weeks.

### 2.3 Follow-up and outcomes

**Figure 4:**
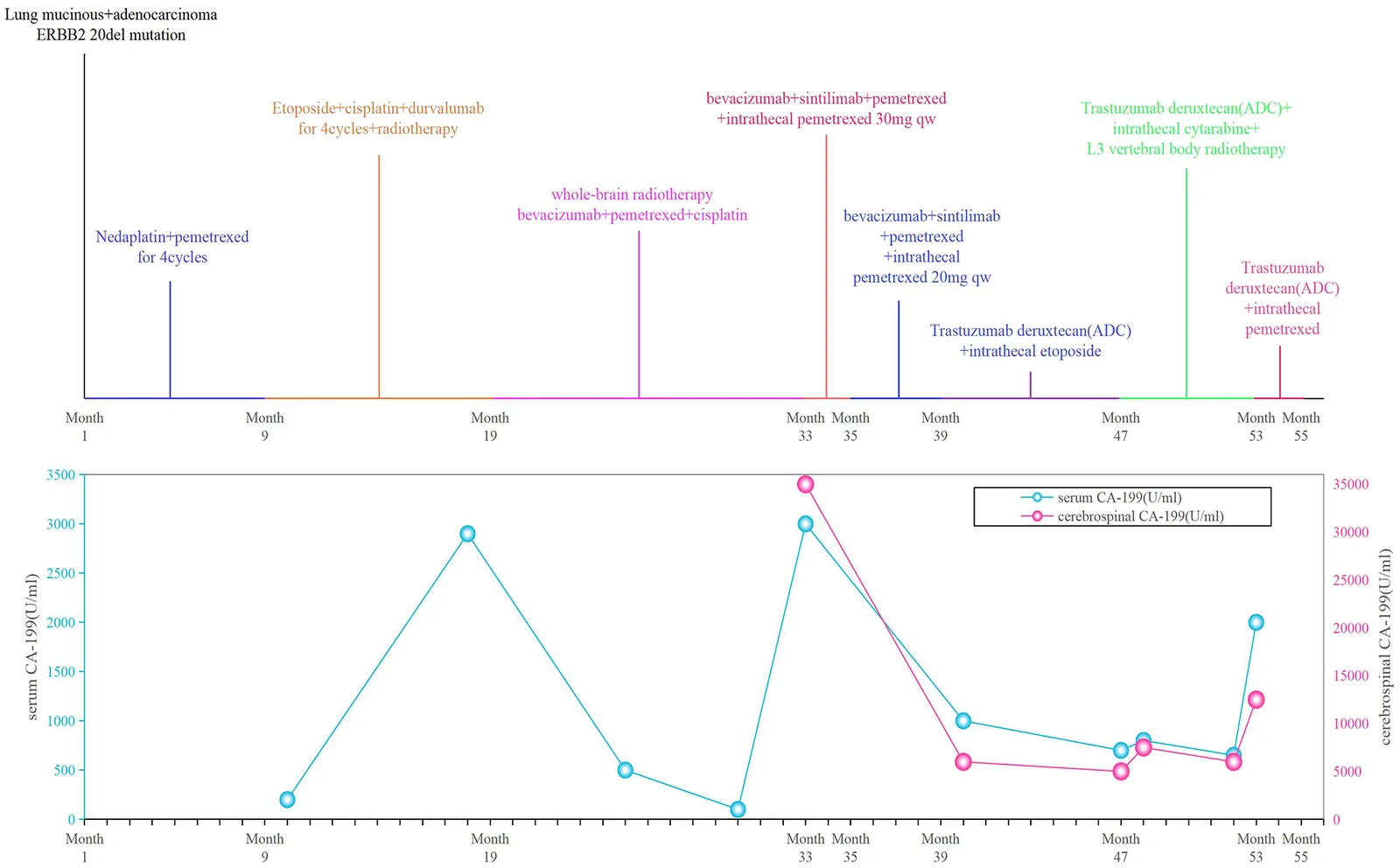
Deidentified clinical timeline and dynamic changes in serum and cerebrospinal fluid CA19–9 levels. Month 1 was defined as the month of initial diagnosis. All subsequent treatments, disease assessments, and biomarker measurements are shown according to deidentified months from Month 1. CSF, cerebrospinal fluid; LM, leptomeningeal metastasis; T-DXd, trastuzumab deruxtecan.

During the latest follow-up in the 55th month, the patient was still alive. Following the clinical diagnosis of LM in the 19th month, her survival duration post-diagnosis has surpassed 36 months (**Figure 4**, **Table 1**). The patient indicated that, despite therapy-induced leukopenia and hepatic impairment, illness management had enhanced her overall quality of life, and she maintained optimism toward future treatment.

**Table 1:** Key timeline of systemic CSF compartment assessment and management.

Time point	Key finding	Management	Interpretation
Month 1	Resected stage IB mucinous lung adenocarcinoma; primary tumor NGS showed ERBB2 p.G776delinsVC, VAF 19.42%	Surgery and adjuvant chemotherapy	Baseline ERBB2-mutant tumor profile established
Month 19	Spinal MRI abnormalities, and atypical cells in CSF	Craniospinal irradiation	Clinically diagnosed with LM
Month 33	No clear systemic progression, but CSF CA19–9 increased to 34,628.4 U/mL; CSF NGS showed the same ERBB2 mutation, VAF 54.26%	Intrathecal pemetrexed	Intrathecal pemetrexed
Month 47-51	L3 metastasis and later recurrent CSF CA19–9 elevation without clear radiographic progression	L3 radiotherapy, intrathecal cytarabine, then pemetrexed rechallenge	Treatment adjusted according to systemic and CSF compartment assessments
Month 55	Patient alive >36 months after LM diagnosis	Continued monitoring	Prolonged survival after multimodal compartment-specific management

CSF, cerebrospinal fluid; LM, leptomeningeal metastasis; MRI, magnetic resonance imaging; NGS, next-generation sequencing; VAF, variant allele fraction.

## 3 Discussion

In this patient, CSF NGS performed at month 33 identified an ERBB2 p.G776delinsVC mutation, consistent with that found in the primary lung tumor; however, the variant allele fraction was significantly greater in CSF than in the primary lung tissue (54.26% vs. 19.42%). This discovery indicates a potential accumulation of ERBB2-mutant clones in the CSF and corroborates the existence of spatial heterogeneity between central nervous system and extracranial disease sites. Prior research indicates that, in individuals with NSCLC and LM, CSF circulating tumor DNA may exhibit superior detection rates and increased abundance compared to plasma circulating tumor DNA, potentially uncovering genetic changes not identified in plasma (13). CSF NGS may yield significant molecular evidence of leptomeningeal involvement and furnish clinically pertinent information to inform subsequent therapy decisions.

The clinical diagnosis of LM is sometimes difficult due to the non-specific of neurological symptoms, and MRI exhibits inadequate sensitivity for early or microscopic leptomeningeal illness. CSF cytology exhibits good specificity; nonetheless, a negative result does not conclusively rule out LM. The EANO–ESMO recommendations advocate for a comprehensive diagnostic strategy that includes clinical presentation, neuroimaging results, and CSF analysis (14). In recent years, the supplementary function of CSF tumor markers in LM originating from NSCLC has garnered heightened interest. A prior study indicated that utilizing a ±25% change threshold for CSF tumor marker levels may more accurately forecast response, progression, and stable illness as delineated by EANO–ESMO criteria (15). This threshold was not formally applied to the present patient because CSF sampling intervals were not prospectively standardized, serial assessment focused primarily on CA19–9 rather than a multi-marker panel, and multiple treatments overlapped between measurements.While these levels necessitate additional validation, they offer a prospective paradigm for longterm surveillance in patients with LM.

CA19–9 is frequently utilized as a supplementary biomarker for the diagnosis and monitoring of treatment in gastrointestinal malignancies, especially pancreatic and biliary tract tumors. Nonetheless, CA19–9 may also be increased in certain lung adenocarcinomas, especially mucinous lung adenocarcinoma, and may indicate tumor load and prognosis (16). In this patient, serum and CSF CA19–9 showed different magnitudes and temporal patterns, raising the possibility of discordant systemic and leptomeningeal disease activity. Nonetheless, the identified correlation does not confirm that CSF CA19–9 more precisely reflected LM activity or autonomously forecasted progression or reaction.

Immune checkpoint inhibitions(ICIs) may have contributed to disease control in this patient, although its specific effects on LM and CSF CA19–9 remain uncertain.In an NSCLC specific cohort, ICIs achieved neurological symptom control in 62.5% of patients with LM, suggesting that selected patients may derive benefit (17).More recently, an international cohort of 2,052 patients with NSCLC and leptomeningeal disease showed improved outcomes and an association between ICIs use and longer LM survival in the subgroup without actionable genomic alterations; however, this finding cannot be directly extrapolated to ERBB2-mutant disease (9). Moreover, available studies mainly evaluated serum tumor markers rather than CSF CA19-9 (18). Therefore, association between immunotherapy and changes in CSF CA19–9 remains speculative and should be interpreted alongside neurological findings, neuroimaging, CSF cytology, and systemic disease status.

The choice of ITC treatment in this instance exemplifies adaptive decision-making in a practical therapeutic environment. ITC can bypass the blood–CSF barrier, enhancing medication exposure in the CSF-containing subarachnoid space; frequently utilized drugs comprise methotrexate, cytarabine, and pemetrexed. A prior phase I research demonstrated that intrathecal pemetrexed was viable for refractory LM from NSCLC, with typically acceptable toxicity when patients were administered folic acid and vitamin B12 supplementation (19). Following intrathecal pemetrexed administration, the patient experienced hepatic dysfunction and myelosuppression, leading the treatment team to transition to etoposide as the intrathecal agent. It was not triggered by an increase in CSF CA19-9. Etoposide induces cytotoxicity by inhibiting topoisomerase II. Fleischhack et al. delivered intrathecal etoposide at a dosage of 0.5 mg daily for five consecutive days, resulting in CSF peak concentrations significantly above those attained with intravenous therapy, while maintaining acceptable tolerability (20). Park documented a patient with NSCLC LM who had a sustained response to salvage intrathecal etoposide following methotrexate failure, which included the resolution of ependymal enhancement in the lateral ventricle, with disease control persisting for almost 19 weeks (21). In our patient, CSF CA19–9 levels remained consistent for several months with intrathecal etoposide therapy, and these data offer a pharmacological and clinical justification for its personalized application, yet do not confirm efficacy in ERBB2-mutant NSCLC. Cytarabine is a conventional agent utilized for intra-CSF therapy of LM; nonetheless, existing guidelines reveal a lack of agreement on the ideal agent, dosage, frequency, and duration. At Month 51, a pemetrexed rechallenge was initiated following a comprehensive evaluation of the patient’s prior apparent response, current neurological and imaging results, systemic disease condition, treatment tolerance, and the absence of a recognized alternative intrathecal regimen. The elevation of CSF CA19–9 was seen as corroborative evidence necessitating reevaluation instead of being viewed as conclusive advancement.Due to the overlap of systemic therapy, radiation, and other intrathecal medicines, the clinical impact of any single intervention remains indeterminate.

The DESTINY-Lung01 and DESTINY-Lung02 trials exhibited sustained clinical efficacy of trastuzumab deruxtecan in ERBB2-mutant NSCLC, with exploratory analysis also confirming its intracranial efficacy in patients with brain metastases (22, 23). LM exhibits distinct biological and pharmacological characteristics compared to parenchymal brain metastasis, and the penetration of large-molecule ADCs into CSF is limited. Consequently, intrathecal delivery of HER2-targeted therapies may necessitate additional research in individuals with HER2-positive LM. A prior case report detailed disease management following intrathecal trastuzumab in conjunction with methotrexate in a patient with ERBB2-mutant NSCLC with LM (24). In HER2-positive breast cancer with LM, intrathecal trastuzumab has demonstrated favorable tolerability and may correlate with extended survival (25). Nonetheless, substantial safety evidence for the intrathecal administration of ADCs is still absent. Clinical trials are required to assess the safety and therapeutic effectiveness of intrathecal HER2-targeted therapies, including ADCs, in patients with ERBB2-mutant NSCLC LM.

Documented cases of ERBB2-mutant NSCLC accompanied with LM are confined to sporadic case reports, and the described treatment strategies have differed significantly. Fan et al, reported a case of a patient with a HER2 exon 20 insertion identified in primary tissue, plasma, and CSF who exhibited swift neurological enhancement following treatment with poziotinib, despite a documented PFS of around 2 months (26). Ghosh et al, later documented a case with a patient administered intrathecal trastuzumab, methotrexate, and hydrocortisone alongside ongoing intravenous pemetrexed. Clinical enhancement was noted during the initial week, CSF cytology turned negative after seven intrathecal administrations, and the patient sustained a PFS at 12 months (24). A separate case report detailed the choice of trastuzumab deruxtecan for HER2 exon 20-mutant and HER2-amplified NSCLC with LM, although post-treatment outcome data were absent at the time of publication (27). Nevertheless, variations in patient attributes, disease severity, previous treatments, systemic disease management, follow-up periods, and simultaneous therapies hinder significant determinations about the superiority of any treatment approach. These reports collectively demonstrate the variability of personalized management in this uncommon clinical context rather than establish a uniform treatment strategy.

The complexity of acquired resistance in NSCLC, as investigated in EGFR-TKI-resistant models, underscores the challenges of long-term disease control in molecularly distinct subtypes (28). The treatment team adopted an integrated strategy in which systemic and leptomeningeal disease were assessed separately but managed in parallel.The systemic disease was evaluated mainly using RECIST 1.1 and radiographic alterations, and was managed with systemic treatments, including chemotherapy and trastuzumab deruxtecan. Leptomeningeal disease was primarily managed with ITC, while serial CSF CA19–9 was recorded as one component of a multimodal longitudinal assessment. When systemic disease appeared stable but CSF CA19–9 increased, the finding prompted closer neurological, neuroimaging, and CSF reassessment for possible leptomeningeal disease activity. Upon the advancement of the L3 vertebral lesion, local radiotherapy was incorporated into the treatment of the bone metastasis, and the ITC regimen was concurrently modified, while the existing ADCs-based systemic therapy was maintained to prevent the premature cessation of an otherwise efficacious systemic regimen. In this instance, sustained disease management did not depend simply on the relentless increase of treatment intensity, but rather on a dynamic equilibrium between efficacy and toxicity.

This case provides a practical example of integrating systemic and leptomeningeal disease management in ERBB2-mutant lung adenocarcinoma. Rising CSF CA19–9 in the setting of stable extracranial disease may raise suspicion for leptomeningeal disease activity, but should not by itself define progression or trigger a change in treatment modification.For patients with LM who achieve prolonged survival, treatment decisions often require continuous balancing of disease control, treatment-related toxicity, patient preference, functional status, and quality of life. In this context, multimodal monitoring may support more individualized and adaptive treatment strategies. The clinical utility of CSF CA19–9 remains exploratory and requires validation in additional cases and prospective cohorts.

However, this study still has several limitations.This report describes a single patient, and the findings cannot be generalized to all patients with ERBB2-mutant lung adenocarcinoma and LM. The selection, dose, and timing of ITC drugs were determined by clinical judgment and biomarker trends, and the efficacy and safety of etoposide in LM associated with NSCLC necessitate validation in larger cohorts. A variety of systemic therapies were provided, with some interventions occurring concurrently. Consequently, the extended survival and alterations in neurological, radiographic, or biomarker indicators cannot be ascribed to any singular therapy. Serial quantitative genomic assessment was not accessible. CSF NGS was conducted at a single documented LM time point, with primary tumor and CSF specimens collected from disparate anatomical regions and at varying phases of the disease. Consequently, the elevated ERBB2 variant allele fraction in CSF must be taken with caution, and the longitudinal dynamics of variant allele frequency(VAF), resistance changes, and clonal evolution could not be evaluated. CA19–9 levels may be affected by histological subtype, inflammation, and overall tumor load. CSF CA19–9 should not be utilized as an independent criterion for assessing progression or response, and its therapeutic applicability necessitates further validation through additional cases and prospective research.

## 4 Conclusion

This report describes a patient with ERBB2 exon 20-mutant mucinous lung adenocarcinoma who received multimodal treatment following the clinical diagnosis of LM. The therapeutic approach comprised a HER2-targeted ADCs, sequential ITC, and localized radiation. The treatment protocol was adaptively modified based on sequential CSF CA19–9 levels, imaging results, and treatment-associated toxicities. The patient has survived for nearly 36 months since the clinical diagnosis of LM and the commencement of treatment, and continues to be monitored. This article details a solitary patient who had several concurrent therapies, making it impossible to ascertain the predictive significance of CSF CA19–9 or the distinct impact of any particular treatment.CSF CA19–9 need to be considered an experimental biomarker instead of an independent instrument for determining illness advancement or directing therapy. Future investigations utilizing standardized CSF collection, established biomarker thresholds, and comprehensive clinical, radiographic, and cytological response criteria are necessary prior to its integration into mainstream clinical practice.

## Data Availability

The original contributions presented in the study are included in the article/supplementary material. Further inquiries can be directed to the corresponding author.
